# Effect of bariatric surgery on future general surgical procedures

**DOI:** 10.4103/0972-9941.78342

**Published:** 2011

**Authors:** Subhash Kini, Umashankkar Kannan

**Affiliations:** Metabolic, Endocrine and Minimally Invasive Surgery, Mount Sinai School of Medicine, New York, USA; 1 Department of Surgery, All India Institute of Medical Sciences, New Delhi, India

**Keywords:** Bariatric surgery, biliopancreatic diversion, obesity procedures, Roux-Y gastric bypass

## Abstract

Bariatric surgery is now accepted as a safe and effective procedure for morbid obesity. The frequency of bariatric procedures is increasing with the adoption of the laparoscopic approach. The general surgeons will be facing many more of such patients presenting with common general surgical problems. Many of the general surgeons, faced with such situations, may not be aware of the changes in the gastrointestinal anatomy following bariatric procedures and management of these clinical situations will therefore present diagnostic and therapeutic challenges. We hereby present a review of management of few common general surgical problems in patients with a history of bariatric surgery.

## INTRODUCTION

**Figure 1:**
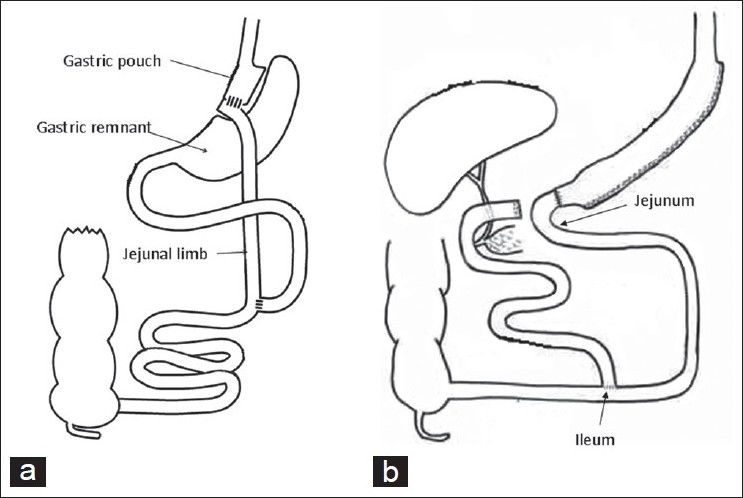
Change in gastrointestinal anatomy following Roux-en Y gastric bypass (a) and biliopancreatic diversion (b).

The laparoscopic approach to obesity surgery has resulted in the increase of bariatric procedures. In the United States, it has increased from 13,000 procedures in 1998 to more than 200,000 procedures per year in 2006.[1,2] As the group of patients with a prior history of bariatric surgery is expanding, it is inevitable that the surgeons are going to see this expanding pool of post-bariatric surgery patients presenting with other common surgical problems in the future. Roux-en Y gastric bypass (RYGB) and the biliopancreatic diversion (BPD), bypass most of the stomach, duodenum and jejunum, altering the gastrointestinal anatomy [Figure 1]. The general surgeons, many of whom have not performed bariatric surgeries, should be aware of these changes in the anatomy and the effects of such changes in future surgical procedures, to diagnose and plan their treatment in this cohort of patients. We, hereby present a review of the management of common surgical procedures in post-bariatric surgery patients.

## MANAGEMENT OF CHOLELITHIASIS AND CHOLEDOCHOLITHIASIS

Rapid weight loss, cholesterol super-saturation in bile, gallbladder dysmotility following division of the hepatic branch of the vagus nerve, absence of duodenum-induced gall bladder emptying — all increase the risk of gallstone formation following a bariatric procedure.[3] Therefore, concomitant cholecystectomy with a bariatric procedure was advocated by few surgeons, to avoid the risk of a second procedure. However, such combined procedures have been found to be associated with increased operating time and hospital stay.[4] Furthermore, in the follow-up of bariatric patients with asymptomatic gall stones, the subsequent cholecystectomy rate for symptomatic patients ranges from 3.8 to 11.5%.[5] Thus, deferring cholecystectomy only in symptomatic patients appears to avoid numerous unnecessary cholecystectomies.[6] In symptomatic patients, laparoscopic cholecystectomy was technically demanding with higher operating time (75 minutes vs 45 minutes) and conversion rate (17.6 vs 1.1%) in comparison to the general population.[7]

The rate of choledocholithiasis following bariatric surgery is 0.4% and the inaccessibility of the biliary tree due to alterations in the anatomy following Roux-en Y gastric bypass and biliopancreatic diversion poses technical dilemmas.[8] New novel methods have been tried and their success in accessing the biliary tree range between 55 and 84%.[9] The different techniques reported are laparoscopic or open transgastric / transenteric endoscopic retrograde cholangiopancreatography, Percutaneous transgastric / transenteric endoscopic retrograde cholangiopancreatography, Percutaneous transhepatic stone extraction from common bile duct, laparoscopic / open common bile duct exploration and retrograde endoscopy.[10] The availability of special skills and the location of stones determine the preferred technique.

Laparoscopic or open transgastric endoscopic retrograde cholangiopancreatography is a novel technique requiring a highly skilled laparoscopic surgeon and endoscopist working in tandem, with a reported success rate of 80%.[11] Entry into the distal stomach remnant can be achieved by either a trocar (15 mm) gastrostomy or by using ultrasonic shears.[11–13] The stomach is then held into the abdominal wall by either suturing it to the fascia or lowering the pneumoperitoneum (7 – 8 mm) or by configuring a tobacco pouch, to avoid leakage of the gastric contents. After introducing the endoscope through the 15 mm trocar, the endoscopist takes over, to carry out endoscopic retrograde cholangiopancreatography and retrieve the stones. The rendezvous technique of catheterizing the biliary tree makes the procedure quicker and safer.[14] A four meter long guide wire is advanced laparoscopically through a tiny incision in the cystic duct and viewed, coming out through the papilla. The guide wire is then caught with the polypectomy snare and the endoscopic retrograde cholangiopancreatography catheter is advanced over the guide wire. In case of biliopancreatic diversion with distal gastric resections and a long roux loop of the intestine, the biliary tree can be accessed through an enterotomy, distal (40 cm) to the Ligament of Treitz, with a standard duodenoscope into the biliopancreatic limb through the umbilical port. Leaving a 26F catheter in the remnant stomach as a tube gastrostomy allows for future access to the biliary tree, in cases of incomplete clearance and to monitor for delayed bleeding following sphincterotomy.[15] Laparoscopy assisted endoscopic retrograde cholangiopancreatography also helps to rule out other potential causes of abdominal pain like internal hernia, marginal ulcers, ulcer within the duodenum and remnant stomach.

In percutaneous transgastric endoscopic retrograde cholangiopancreatography, a pigtail catheter is inserted under ultrasound or computed tomography guidance. It is then dilated gradually every one or two weeks to the desired size (14Fr to 20 or 24 Fr) to introduce the endoscope.[16] It is technically difficult as the remnant stomach cannot be distended with air. Furthermore, as it requires serial dilations, it cannot be employed in emergent situations like cholangitis, where laparoscopy provides a quicker access.[13] Fobi *et al*.,[17] recommended routine placement of a gastrostomy tube with a silastic radiological marker in the remnant stomach during the index surgery, for later access. However, such routine placements cannot be justified due to a lower incidence of choledocholithiasis, following Roux-en Y gastric bypass.

Percutaneous transhepatic cholangioscopy is introduced for treatment of residual bile duct stones. Standard percutaneous transhepatic cholangioscopy is done by using the Burhenne’s technique, wherein common bile duct stones are removed with a steerable catheter and basket, under fluoroscopic guidance. In bariatric patients, this technique may be preferred in those cases where intrahepatic ducts are well dilated and cholecystectomy had been done previously.

Laparoscopic or open common bile duct exploration through the transcystic route or choledochotomy is an established modality for clearance of common bile duct stones. It may be preferred if the duct is dilated and other modalities have failed. It also enables direct visualisation of the biliary tree by intraoperative cholangiography and choledochoscopy, to ensure clearance. In case of incomplete clearance due to impacted stones or after repeated attempts, choledochoduodenostomy can be also attempted if required. A T-tube can be placed for later cholangiography, choledochoscopy, lithotripsy, stone extraction by flushing, basket or by dissolution therapy in case of incomplete clearance. However, to our knowledge, the use of this technique has not been reported in post-bariatric surgery patients so far.

Retrograde endoscopy is another novel method to access the biliary tree after bariatric surgery. Access can be achieved either with a standard duodenoscope, a pediatric colonoscope, or an enteroscope, depending on the length of the Roux limb. The pediatric colonoscope and the double balloon enteroscope follow a tortuous route through the oesophagus, gastric pouch, efferent limb, jejunojejunostomy, afferent limb, duodenum, and finally, the distal gastric remnant. Retrograde gastric remnant intubation is successful with a pediatric colonoscope in 65% of the cases and with a double balloon enteroscope with an overtube in 83% of the cases.[18] With the increasing length of the Roux and biliopancreatic limbs, the success rate is expected to fall.

## MANAGEMENT OF COMPLICATED DUODENAL ULCERS

The diagnosis of peptic ulcer following the Roux-en Y gastric bypass and biliopancreatic diversion presents several challenges. Histological confirmation of *H. pylori* remains the diagnostic gold standard and requires endoscopic access. However, endoscopic access and examination of the remnant stomach and duodenum requires expertise in endoscopy, laparoscopy, and radiology, as discussed in the management of choledocholithiasis. The urea breath test is non-invasive, but shows false negative results, as urea does not come in contact with the *H.pylori* in the distal stomach and duodenum. Stool antigen detection with a sensitivity and specificity of 91 and 93%, respectively, is probably the best non-invasive test for confirming the presence of *H. pylori*.[19]

The rate of duodenal and gastric perforations in open Roux-en Y gastric bypass is 0.26%.[20] *H. pylori* plays a prominent role in ulcerogenesis in this cohort of patients.[21] Also reported is necrosis and perforation of the biliopancreatic limb, secondary to obstruction and dilation. Out of the 23 cases reported, air under the diaphragm has been reported in only one of the cases.[22–24] This is because the air in the bypassed remnant stomach is absorbed in the post-operative period, except in cases of gastrogastric fistula and an obstruction distal to an enteroenterostomy. Hence, it is important for the physician to understand that perforation in bariatric surgery patients can present without the usual clinical and radiological signs. Primary closure with an omental pad is reported to have a high re-operative rate, with nine out of eleven cases requiring subsequent surgery in a series.[20] The probable reasons for re-operation with primary closure may be due to acidity with a mean pH of 2 – 3 recorded in the distal gastric remnant.[25] Normal response to vagal stimuli in the remnant stomach, absence of buffering by the ingested food, bile reflux, abnormal pancreatic secretion — all these contribute to the acidic environment. Definitive surgery for perforation is the total excision of the bypassed stomach either at presentation or after recovery, following a primary closure. Pancreas preserving the duodenal resection, with non-viable stomach and jejunum, has been reported in a case of perforation secondary to obstruction and dilatation of the biliopancreatic limb. The remnant stomach and duodenum have multiple full thickness necrotic patches with only 1 cm of duodenum around the ampulla of vater viable.[22]

Bleeding from a peptic ulcer after gastric bypass is reported to be at the rate of 0.27%. However, difficulty in accessing the bypassed remnant poses diagnostic and therapeutic challenges. A Techetium-labelled red blood cell scan can help in localising the site in such cases.[26] Resection of the distal stomach is the definitive treatment for bleeding ulcers.[26]

## MANAGEMENT OF OESOPHAGEAL CANCER

**Figure 2:**
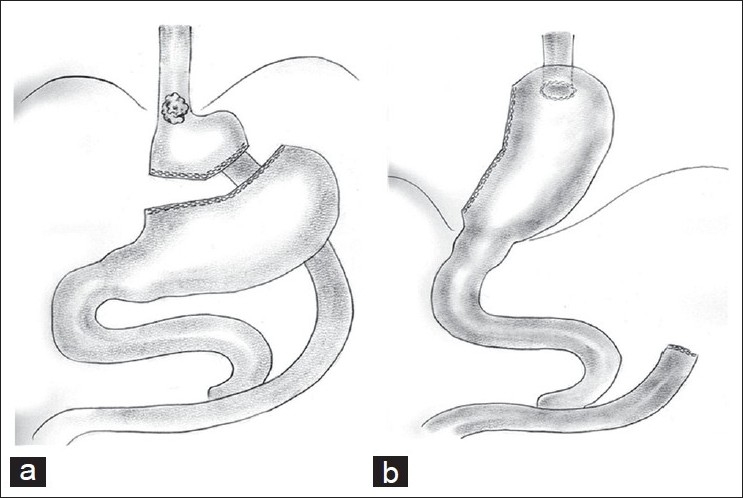
Gastrointestinal anatomy following Roux-en Y gastric bypass (a) and reconstruction (b), by Ivor-Lewis’ technique, for oesophageal cancer. Reproduced with permission from Nguyen *et al*.[29]

Oesophagectomy / oesophagogastrectomy is the preferred treatment for resectable oesophageal cancers in the general population. Roux-en Y gastric bypass, the commonly performed bariatric procedure, alters the anatomy of the stomach and provides technical challenges in using the stomach as a conduit. Use of the gastric remnant and free jejunal free-tissue transfer between the oesophagus and the remnant stomach as conduits are reported in the treatment of aesophageal cancers, following the Roux-en Y gastric bypass.[27–29] Nguyen *et al*.,[29] have reported the use of a minimally invasive Ivor-Lewis technique for a T1N0 lesion [Figure 2]. The use of Intrathoracic anastomosis in the Ivor-Lewis technique minimizes the tension of oesophagogastric anastomoses, as a portion of the stomach had been made use of in creating a pouch during the Roux-en Y gastric bypass procedure. Allen *et al*.,[27] have reported the use of jejunal free-tissue transfer as a conduit with anastomoses to the cervical oesophagus proximally and to the gastric remnant distally. There is no account of the colon being used as an alternative conduit in this group of patients, but evaluation of the colon preoperatively with mesenteric angiograms for a possible use has been reported.[28]

## MANAGEMENT OF PANCREATIC LESIONS

**Figure 3:**
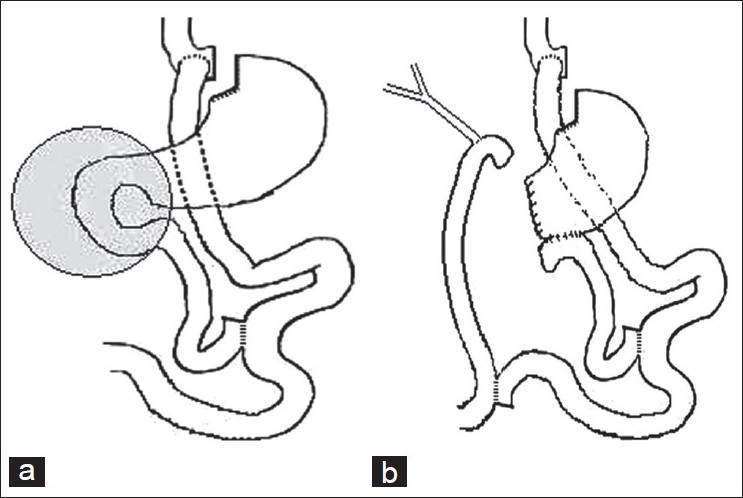
Gastrointestinal anatomy following Roux-en Y gastric bypass (a) and reconstruction (b), after pancreaticoduodenectomy for cancer in the head of the pancreas. Reproduced with permission from Rutkoski *et al*.[30]

**Figure 4:**
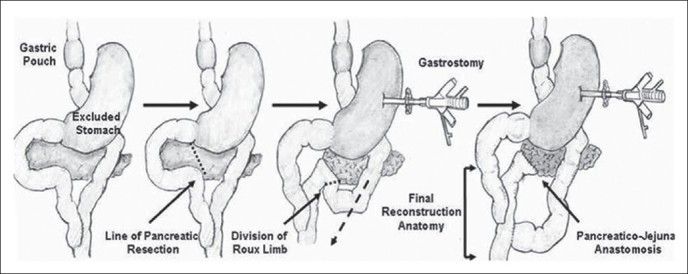
Intestinal reconstruction following the Berger’s procedure. Reproduced with permission from Barbour *et al*.[32]

**Figure 5:**
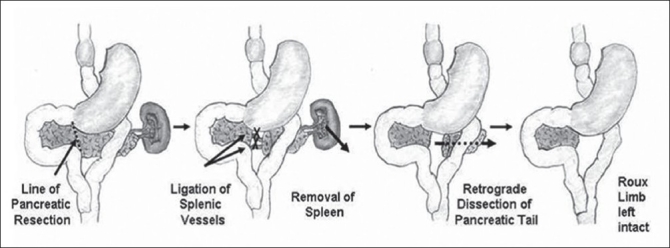
Intestinal reconstruction following distal pancreatectomy. Reproduced with permission from Barbour *et al*.[32]

Pancreatic head cancer manifesting after Roux-en Y gastric bypass presents technical challenges in reconstructing the gastrointestinal anatomy. Rutkoski *et al*.,[30] have reported a case of pancreaticoduodenectomy for a 2 cm mass in the pancreatic head, presenting 3.5 years after Roux-en Y gastric bypass [Figure 3]. Kuper *et al*.,[31] have reported a case of pancreatic cancer following sleeve gastrectomy that was treated with Pylorus preserving pancreaticoduodenectomy for a pT3 tumor, which recurred nine months post-operatively. Barbour *et al*.,[32] have described the Beger’s procedure of duodenum-preserving pancreatic head resection [Figure 4] and distal pancreatectomy with splenectomy / cystectomy [Figure 5] in five patients (two cases of chronic pancreatitis, two cases of Nesidioblastosis and one case of serous cystadenoma) after Roux-en Y gastric bypass. Clancy *et al*.,[33] recommend total or near-total (95% of pancreas) pancreatectomy to avoid recurrence. In post-Roux-en Y gastric bypass patients, a piecemeal resection of the pancreas with pancreatic tissues, to the left of the Roux limb removed first and then on the right of the Roux limb is described. The extent of pancreatectomy in nesidioblastosis is still controversial. It has been shown that the likelihood of cure increases with the extent of resection, at the expense of increased risk of exocrine pancreatic insufficiency and insulin-dependent diabetes-mellitus.[34]

## MANAGEMENT OF GASTRIC CANCER

Seven cases of gastric carcinoma, between one and twenty-two years, following Roux-en Y gastric bypass have been seen in literature.[35–41] Six out of the seven cases presented with abdominal pain. Vague symptoms combined with the inability to access the bypassed segment delayed the diagnosis. All resectable cases had been treated with total excision of the remnant stomach. In the unresectable cases, the remnant stomach was decompressed by a gastroenterostomy between the remnant stomach and the Roux limb.[35] Some surgeons advocated the practice of routine gastropexy to the anterior abdominal wall at the time of the index procedure for later access and surveillance.[41]

## MANAGEMENT OF THYROID LESIONS

Duodenum and the proximal jejunum, the predominant sites of absorption of calcium are bypassed in the Roux-en Y gastric bypass and biliopancreatic diversion, placing these patients at risk of hypocalcaemia.[42] Despite normal levels of calcium, vitamin D, parathormone before thyroidectomy and preserving more than two parathyroid glands intra-operatively, these patients can go in for severe hypocalcaemia in the post-operative period.[43] One remarkable feature in these patients is that the parathyroid glands are found to be hypertrophied during surgery. Routine perioperative intravenous 10% calcium gluconate started within one hour after thyroidectomy and a prescription of large doses of calcium, calcitriol and ergocalciferol, with subsequent dose adjustments, may reduce the occurrence of severe life threatening hypocalcaemia.[44]

## MANAGEMENT OF KIDNEY STONES

Following Roux-en Y gastric bypass and biliopancreatic diversion, there is hyperoxaluria and increased incidence of kidney stone formation.[45] Fernandez-Lobato *et al*.,[46] reported dehiscence of the gastrojejunal anastomosis following extracorporeal shock wave lithotripsy for a left renal calculus, with a history of biliopancreatic diversion, four years before. A patient was operated for anastomotic leak one day after lithotripsy and the anastomosis was hand-sewn. The cavitations effect created by lithotripsy could reach the titanium staples in the gastrojejunal anastomosis, heat them and cause tissue damage, resulting in leak, as in this case. Hence, a close surveillance for leak after the procedure is advised, to detect complications early.

With elevated body mass index increasing the risk of allograft failure in renal transplantation and reports of safe bariatric surgery in patients with chronic kidney disease, bariatric surgery is emerging as a potential option for improving the candidacy of patients wait-listed for renal transplantation. Use of dextrose in the dialysis solution and leaving a catheter in peritoneal dialysis raises concerns of risk of infections after surgery with gastric banding, although there are no reports to support or refute the claim. The choice of bariatric surgery and the effect of dialysis in the outcome are still unclear in these patients.

## MANAGEMENT OF ORTHOPAEDIC LESIONS

Roux-en Y gastric bypass, bypasses the duodenum and jejunum, the major sites of calcium absorption. This limits the absorption to only about 20% of dietary calcium in the remaining intestine and is dependent on Vitamin D. However, Vitamin D, being a fat-soluble vitamin is also poorly absorbed due to uncoordinated, poor mixing of bile and pancreatic secretions with the food. The body compensates for this hypocalcaemia by upregulating the parathormone, which increases bone resorption. Hence, in patients with bypass history, considered for orthopaedic surgery, the orthopaedic surgeons should be prepared for poor bone quality resulting in delayed fracture healing and non-union of an arthrodesis.[47] Hey *et al*.,[48] report a case of non-union of a distal radius fracture following a minor trauma, in a patient with a history of jejunoileal bypass, five years earlier. The fracture healed and biochemical abnormalities normalised after the intestinal re-anastomosis.

## CONCLUSION

With such expanding effects of bariatric surgery on other surgical diseases, surgeons need to be prepared to handle these challenging cases. General surgeons working with a multidisciplinary team can improve the outcome in these patients.
